# Bat songs as acoustic beacons - male territorial songs attract dispersing females

**DOI:** 10.1038/s41598-017-14434-5

**Published:** 2017-10-24

**Authors:** Mirjam Knörnschild, Simone Blüml, Patrick Steidl, Maria Eckenweber, Martina Nagy

**Affiliations:** 1 Animal Behavior Lab, Free University Berlin, Takustr. 6, 14195 Berlin, Germany; 2 Smithsonian Tropical Research Institute, Barro Colorado Island, Roosevelt Ave., Tupper Building - 401, Balboa, Ancón, Panama City, Panama; 3 Institute of Experimental Ecology, University of Ulm, Albert-Einstein-Allee 11, 89069 Ulm, Germany; 4 Museum für Naturkunde - Leibniz Institute for Evolution and Biodiversity Science, Invalidenstraße 43, 10115 Berlin, Germany

## Abstract

Male song in birds and mammals is important for repelling rivals, stimulating mates or attracting them to a specific location. Nevertheless, direct experimental evidence for the mate attraction function of male song is limited to a few studies. Here, we provide strong experimental evidence that male songs attract wild female bats (*Saccopteryx bilineata*). Playbacks of territorial songs reliably elicited phonotaxis in females but not males. Most females captured during playbacks were subadults searching for new colonies to settle in. In *S. bilineata*, multiple males sing simultaneously at dawn and dusk, thereby creating a conspicuous chorus which encodes information on colony identity and size. Since territorial songs have a large signalling range, male songs constitute acoustic beacons which enable females to localize new colonies. In our playbacks, females strongly preferred local territorial songs over foreign territorial songs from two different locations, indicating that song familiarity influences phonotaxis. Our study provides the first clear experimental evidence that male song elicits female phonotaxis in a non-human mammal. Bats are an especially promising taxon for studying mammalian song since male song has been described in different species with diverse social organisations and natural histories, thus providing exciting opportunities for phylogenetically controlled comparative studies.

## Introduction

Birdsong is considered to be crucial for rival deterrence, mate attraction and stimulation (reviewed in refs^1–3^). Singing males can achieve these effects either by (1) using the same song characteristics for male and female receivers or by (2) changing certain aspects of their singing behaviour or (3) producing different song types when interacting with males versus females (reviewed in refs^4,5^). Indirect correlative evidence for the dual function of birdsong, rival deterrence and mate attraction / stimulation, is ubiquitous (reviewed in refs^5–9^). Moreover, there is ample direct experimental evidence that birdsong repels rivals: muted males lose their territories^10,11^, take-overs of vacant territories can be delayed by a speaker broadcasting song^12–14^, and territorial males approach and attack speakers broadcasting conspecific song within their territories^15–17^. There is also direct experimental evidence that birdsong stimulates females: male song elicits a copulation solicitation display in many female passerine birds^18,19^ and high quality male song prompts receptive females to build nests faster and lay more eggs than low quality song^20^. Moreover, many playback experiments have demonstrated that females prefer hearing familiar song over unfamiliar song^21–23^. However, direct experimental evidence that male song attracts females to a specific location, i.e. phonotaxis, is surprisingly rare, probably because it is difficult to disentangle the use of song to obtain a territory (and thus have potential access to females) and the use of song to actually attract females to the territory (reviewed in refs^2,3,5^). As a consequence, only a few studies, all conducted in the wild, attempted to test experimentally whether male song elicits phonotaxis in female birds (pied and collared flycatchers^24^, sage grouses^25^, European starlings^26^, house wrens^27^, and hoopoes^28^).

Understanding the multiple functions of male song in birds and in other singing species is a crucial endeavour since the function of a behavioral trait enables us to infer the context in which it has evolved^29^. ‘Song’ is an elaborate vocalization with a specific spectro-temporal pattern that is used during courtship or territorial defence^9^; songs differ drastically from the structurally more simple calls which are produced by many male mammals (e.g. roaring in deer or howling in canines). Except for humans, only a few mammalian singers are known, for instance humpback whales^30,31^, gibbons^32,33^, rock hyraxes^34,35^, and bats^36^. Singing bats, order Chiroptera, have only recently attracted in-depth scientific interest and the different functions of bat song are not thoroughly described. Male song has been found in five of the 17 extant bat families to date (reviewed in ref.^36^) but it is highly likely that singing is more widespread considering that social vocalizations have been studied in very few bat species. Playback studies on the function of bat song in the context of mate attraction are still scarce and difficult to interpret since approaching bats were not captured, thus making it impossible to know whether males, females or both sexes approached the song playback^37^.

One of the most thoroughly studied singing bat species is the greater sac-winged bat *Saccopteryx bilineata*. Male song in *S. bilineata* has first been reported in the 1970s^38–40^ and the species’ natural history, social system and communication have been extensively studied in the last two decades (reviewed in refs^41–43^). Male *S. bilineata* defend year-round roosting territories in communal, perennial day-roosts from male competitors. Up to eight females may roost in a single male’s territory^38–40^. Social communication is multimodal, with acoustic and olfactory signals being most prevalent (reviewed in refs^41,42^). Female choice plays an important role since males cannot monopolize or dominate females, which freely select their mating partners from colony males^44,45^. Young females disperse from their natal colony to avoid inbreeding, whereas many young males remain in their natal colony^46^. Male-biased philopatry is also found in songbirds^47^.

**Figure 1:**
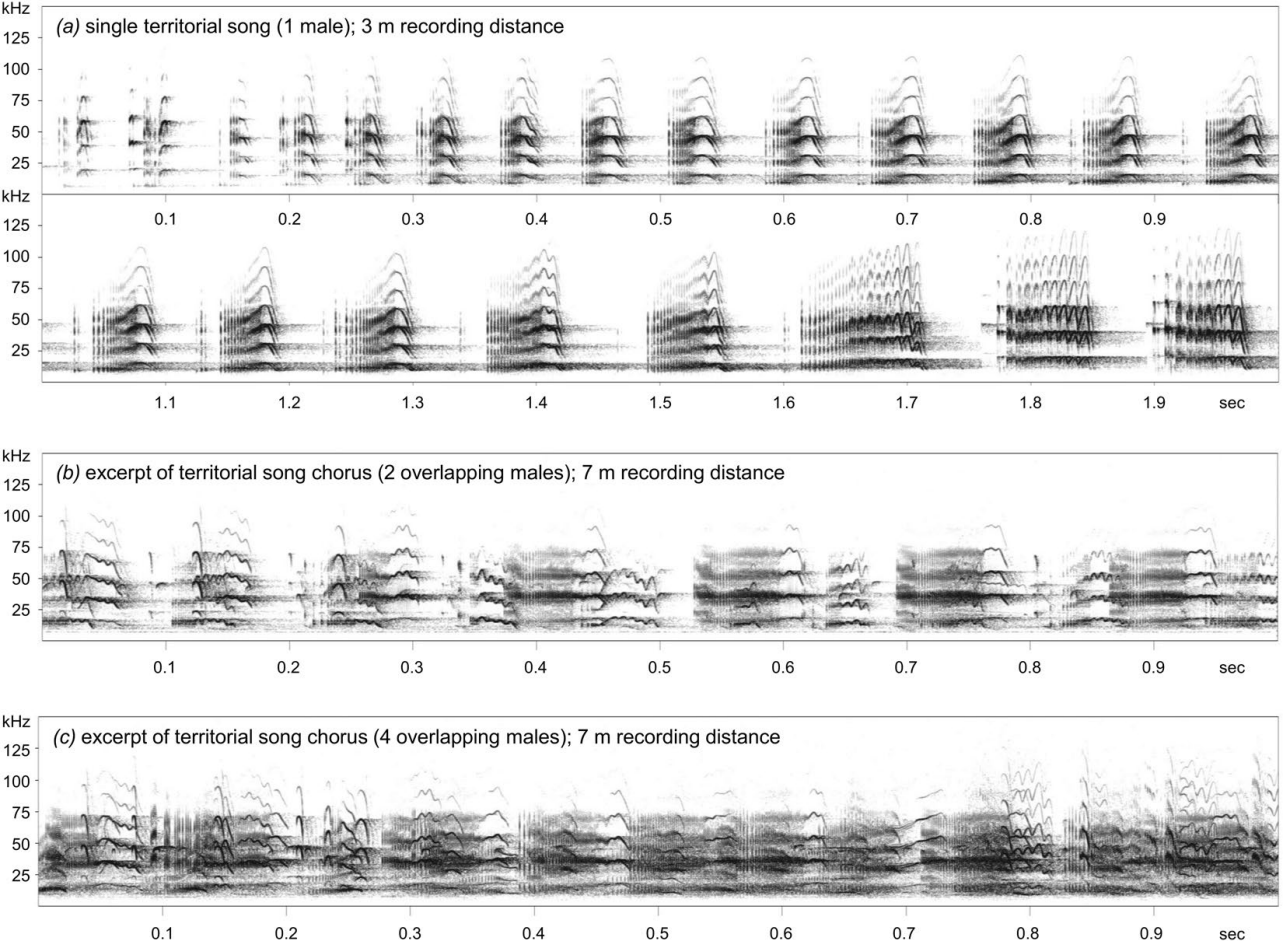
Single territorial song (**a**) and territorial song chorus excerpts (**b**,**c**) from *S. bilineata* males. The two song chorus excerpts were recorded at small (**b**) and large (**c**) day-roost colonies with two and four singing males overlapping, respectively. The single territorial song was recorded at a distance of three meters, both chorus excerpts were recorded at a distance of seven meters. Spectrograms depict frequency over time and were generated using a Hamming window with 1024-point fast Fourier transform and 87.5% overlap.

Male *S. bilineata* sing throughout the year but singing is most intense prior and during the annual mating period in December. Males produce territorial songs mainly at dawn and dusk (in the 30 minute period after entering the day-roost in the morning and before leaving it in the evening) but also sporadically at any time during the day^48^. Territorial songs are multisyllabic vocalizations with a specific sequential structure. They start with simple tonal syllables that gradually merge into composite buzz syllables which consist of a harsh, pulsed part and a tonal part^48^ (see Fig. 1a). Depending on the agitation level of the singing bat, the tonal part of buzz syllables can be moderately to highly modulated. Trill-like modulations indicate aggressive interactions in the near future^39^. Territorial songs have an average length of 1.6 seconds (maximum: 4 seconds) and buzz syllables an average peak frequency of 14.5 kHz (minimum: 7 kHz), which is comparatively long and low frequency for a bat and clearly audible for a human listener^49–51^. Buzz syllables encode an individual signature^50,52,53^ and a group signature^52^, thus providing specific information about the signaller. During vocal ontogeny, pups learn territorial songs by imitating the song of adult tutors^54^. Vocal imitation, along with genetic drift, can lead to regional variation of song syllables^53^.

Even though territorial songs are used for mediating territorial disputes among males, it is conceivable that they can also be directed at females or that females gain valuable information by eavesdropping on vocal exchanges between males. The dawn chorus of singing *S. bilineata* males is a widely audible vocal display and thus strongly resembles the dawn chorus of songbirds. All territorial males from a day-roost colony simultaneously produce territorial songs at dawn, which makes day-roosts highly conspicuous, especially when many singing males contribute to the dawn chorus. As in songbirds, *S. bilineata’s* dawn chorus may not only repel rivals^49,50^ but also attract females, specifically subadult females. All subadult females disperse prior to reproduction at the age of three months to new colonies to avoid inbreeding^46^. Females are known to disperse within the area they were born in (in our study populations, we observed dispersal distances of up to three kilometres), suggesting that females may still have access to familiar foraging grounds after dispersal.

The dawn chorus could be of particular importance for these dispersing females as it may help them to localize new colonies in which to immigrate. Since we, as researchers, are able to locate previously unknown day-roost colonies in the forest by listening to *S. bilineata’s* dawn chorus and following the songs to their sources, it is possible that dispersing females are able to do the same. Correspondingly, roost finding is facilitated by conspecific vocalizations in several bat species^55–60^ but these vocalizations are neither male-specific nor do they elicit phonotaxis in females only.

Moreover, communal choruses in other group-living, territorial birds and mammals can encode information on group size (e.g. green woodhoopoes^61^, lions^62^) or group identity (e.g. laughing kookaburras^63^, wolves^64^), so it is conceivable that chorusing bats may do the same. If the dawn chorus of *S. bilineata* encodes information on colony identity and size, dispersing females could take advantage of this information. They should prefer dispersing to a large colony since this would lead to a greater choice of potential mating partners than dispersing to a smaller colony^65,66^.

In this study, our overall hypothesis was that male territorial songs constitute acoustic beacons which facilitate the natal dispersal of female bats. Specifically, we estimated the signalling range of *S. bilineata*’s territorial songs and tested whether conspecifics are attracted to playbacks of territorial song. We predicted that only females, especially dispersing subadult females searching for new colonies, would exhibit phonotaxis to song playbacks. Moreover, we tested whether female preference depended on the familiarity to song playbacks. We hypothesized that female bats would be more attracted to local territorial songs than to foreign songs from different regions, since female songbirds exhibit a strong preference for familiar songs as well^21–23^. In addition, we analysed the information content of whole song choruses. We hypothesized that bat song choruses encoded information on colony identity and size since this information is also encoded in the communal choruses of other group-living birds and mammals^61–64^.

## Results

### Territorial song chorus functions as acoustic beacon

A previous study on *S. bilineata* measured the amplitude of territorial songs (Fig. 1a) as 96 dB SPL at a distance of one meter^49^. By using a formula originally developed for calculating the maximum detection distance of objects by bat echolocation calls^67^ and adapting it to social vocalizations (see methods for details), we estimated an acoustic signalling range of 124.2 meters for territorial songs in open habitats. However, it is possible that the detection threshold of the receiver, set at 20 dB SPL for echolocation pulses^67^, is lower for territorial songs; this, in turn, would increase the signalling range. We therefore also calculated the signalling range solely based on atmospheric attenuation (detection threshold of receiver set at 0 dB SPL). The estimated theoretical maximum signalling range of territorial songs in open habitats was 184.6 meters. Even though these results are only rough estimates and territorial songs certainly attenuate faster in cluttered forest habitats than in open habitats, the estimated signalling ranges are in line with our personal experience that territorial songs are clearly audible to human listeners in the forest at dawn and dusk.

**Figure 2:**
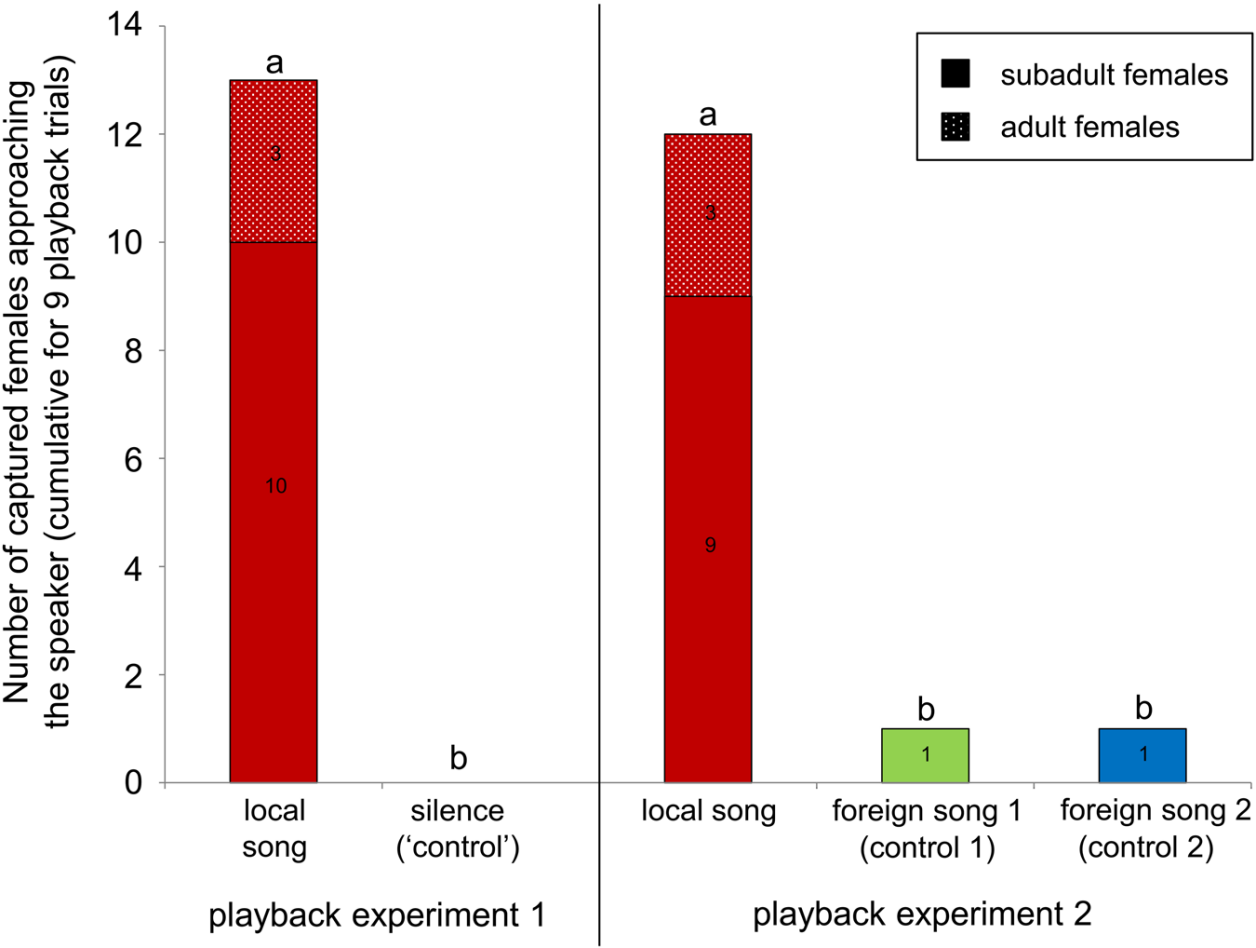
Results of two playback experiments investigating the phonotaxis of wild *S. bilineata* females towards a speaker broadcasting local songs vs. silence (playback 1, 2014) or local songs vs. foreign songs from different regions (playback 2, 2015). Local songs were recorded at our study site BCI in Panama in previous years (2010–2012); foreign songs were recorded at two sites in Costa Rica, Curú and Santa Rosa. The males from which we recorded local songs were not present at our study site anymore and their songs thus unknown to subadult females in our playbacks. Different letters depict a statistically significant difference.

Male song playbacks were broadcasted from currently uninhabited day-roosts at dawn (see Figure S1 in Supplementary Information for details), when territorial males had already returned to their day-roosts while adult females, adult bachelor males (i.e. males without a territory) and subadults of both sexes were still foraging. Each playback trial was conducted at a different site with different male songs; male song donors and the location of playback day-roosts were unknown to subadults of both sexes and, very likely, to adults as well. Our first, preliminary playback experiment (testing broadcasted songs against a silent ‘control’) revealed that females were attracted to territorial songs and not to uninhabited day-roosts without song playbacks (Wilcoxon signed-rank test; Z = −2.739, N = 9, exact P = 0.004; Fig. 2). Males were never attracted to playbacks trials. During nine playback trials, we captured a total of 13 females (10 subadults, 3 adults) while they approached the speaker broadcasting territorial songs. We captured at least one subadult female per playback trial (see Table S1 in Supplementary Information for details). In one trial, we captured two subadult females and in three trials, we captured one subadult female and one adult female each (which were mother-daughter pairs on two occasions). No bats were attracted during trials when no territorial songs were broadcasted (silent ‘control’; no other control stimulus was feasible; see justification in methods).

A second playback experiment was needed to clearly demonstrate that female phonotaxis was stimulus-specific, i.e. could only be elicited by territorial songs and not any other sound. Details of the second playback experiment (testing local songs against foreign songs as control) can be found below.

### Females prefer local territorial songs over foreign songs

**Figure 3:**
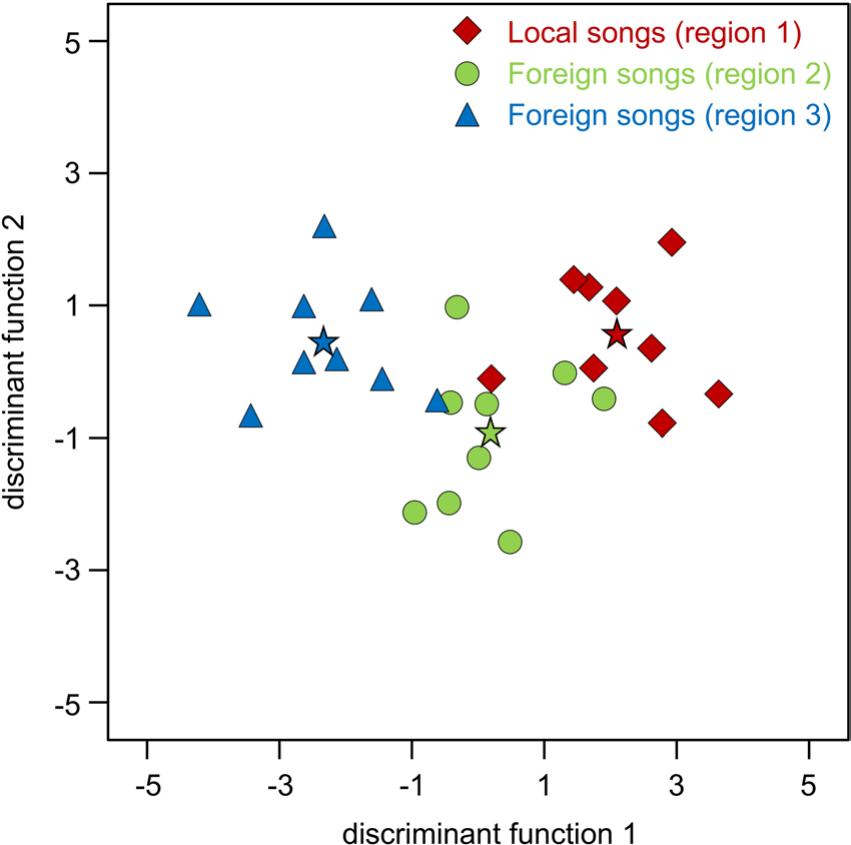
Acoustic signal space obtained by a DFA depicts the relative position (centroids) of 27 *S. bilineata* males from three different regions (region 1: BCI in Panama; region 2: Curú in Costa Rica; region 3: Santa Rosa in Costa Rica) based on their territorial song parameters. Different regions are encoded by different symbols, group centroids by asterisks. Males from the same region cluster together in signal space, indicating that they produced territorial songs with a regional signature.

Territorial songs from different regions differed in their acoustic parameters. A DFA with territorial songs of 27 males from three regions in Panama and Costa Rica (9 males per region, 10 songs per male) classified 74.1% of all males to the correct region (Fig. 3, Table S2 in Supplementary Information; classification success expected by chance was 33.3%).

Correspondingly, our second playback experiment indicated that females were able to perceive the acoustic differences between local territorial songs (recorded at our field site in Panama in previous years) and foreign territorial songs (recorded at two regions in Costa Rica). As in the preliminary playback, male songs were broadcasted from currently uninhabited day-roosts at dawn. Each playback trial was conducted at a different site with different male songs from three different regions; male song donors from the same region and the location of playback day-roosts were unknown to subadults of both sexes and, very likely, to adults as well. Female *S. bilineata* were significantly more attracted to local territorial songs than to foreign territorial songs (Friedman test; χ^2^ = 14.769, N = 9, exact P < 0.0001; Fig. 2). During nine playback trials broadcasting local and foreign territorial songs in a pseudo-randomized order, we captured a total of 14 females (11 subadults, 3 adults) while they approached the speaker (see Table S1 in Supplementary Information for details). The majority of females (12 out of 14) were captured when local territorial songs were broadcasted. We never captured any males.

### Colony-specific information is encoded in the territorial song chorus

Large colonies (with 2–4 singing males) created a different auditory impression than small colonies (with 1–2 singing males). At a recording distance of 7 meters, large colonies tended to produce a louder song chorus than small colonies (54.25 dB SPL vs. 51.98 dB SPL; GLMM: AIC = 60.194, F_1,3.969_ = 5.548, P = 0.079; Fig. S2a in Supplementary Information) but this trend disappeared at a recording distance of 14 meters (49.40 dB SPL vs. 47.58 dB SPL; GLMM: AIC = 71.252, F_1,3.550_ = 3.422, P = 0.148; Fig. S2b in Supplementary Information). A louder song chorus is probably achieved by multiple males singing at the same time, which results in an accumulation of independent sound sources, i.e. singing males (Fig. 1b,c).

Moreover, large colonies could, to a certain degree, be discriminated from small colonies by the different acoustic properties of their song chorus. One out of five derived acoustic parameters (LFCCs 1–5; linear frequency cepstral coefficients) differed significantly between large and small colonies (LFCC 3; GLMM: AIC = −294.7, t_1,317_ = −2.156, P = 0.031; results for other LFCCs were not significant) at a recording distance of 7 meters, but not at a distance of 14 meters (P = n.s. for all five LFCCs; see Table S3 in Supplementary Information for details).

In addition, a colony-specific signature was encoded in the territorial song chorus which could be used to discriminate between different colonies based on chorus acoustics alone. A DFA with 321 chorus excerpts from six colonies (recorded at 7 meters distance) classified 66.5% of all chorus excerpts to the correct colony (Table S4 in Supplementary Information). The colony-specific signature was less pronounced at 14 meters recording distance. Using a similar amount of chorus excerpts from six colonies, a DFA classified 45.3% of all chorus excerpts to the correct colony (Table S4 in Supplementary Information). The correct classification rate expected by chance was 16.7% for both DFAs.

## Discussion

This study provides strong experimental evidence that territorial songs of male *S. bilineata* can attract females, especially dispersing subadult females, to previously unknown colonies. Six out of 21 females attracted to male song playbacks were adults, which were apparently searching for new colonies as well. The results of both playback experiments combined indicate that the observed phonotaxis is stimulus-specific (local songs are strongly preferred over foreign songs) and restricted to females. To our knowledge, this is the first unequivocal experimental evidence that male song elicits female-specific phonotaxis in non-human mammals. Male song might not be the only way for female *S. bilineata* to locate new colonies; females could also follow conspecifics to unknown day-roosts by eavesdropping on their echolocation calls. Nevertheless, our results clearly indicate that territorial songs alone are sufficient to attract females to a specific location. Male vocalizations eliciting female phonotaxis are known from diverse taxa (insects^68^, fish^69^, anurans^68^, birds^24^) but clear experimental evidence for this phenomenon is scarce in birds (reviewed in refs^2,3,5^) and had been lacking for mammals before this study.

Territorial songs of male *S. bilineata* are broadcast over considerable distances and can thus be considered to be acoustic beacons. The amplitude of *S. bilineata’s* territorial songs is comparable to similar sized birds^70,71^: Eurasian wrens, *Troglodytes troglodytes*, and common chiffchaffs, *Phylloscopus collybita*, produce song at 90 dB SPL while coal tits, *Periparus ater*, sing at 78 dB SPL (at a distance of 1 meter, respectively). Signal amplitude is crucial for determining the active space of a communication signal since louder signals travel greater distances and can be better detected by receivers (reviewed in refs^72–74^). Acoustic theory predicts a smaller active space for bat song compared to bird song produced at the same amplitude since the former has a higher peak frequency and thus attenuates faster^74^. Direct comparisons between the active space of bat and bird song are difficult because ambient noise levels and vegetation-caused attenuation can differ drastically and, in most species, the receivers’ thresholds for detecting song have not been studied so far (but see refs^75,76^). Nevertheless, song amplitude can be an important signal in itself. Females of many different taxa prefer intense sound levels of male vocalizations (insects, anurans^77^; birds^78,79^). In *S. bilineata*, large day-roost colonies with many singing males tended to have a louder dawn chorus with a different spectral composition than small day-roost colonies with few singers. This more conspicuous acoustic beacon is most likely caused by the accumulation of multiple independent sound sources, i.e. singing males, during chorusing. However, it is currently unclear whether individual males in large colonies sing louder than males in small colonies since we only measured the amplitudes of whole dawn choruses.

Territorial, group-living birds and mammals also signal group size with their choruses (green woodhoopoes^61^, lions^62^ and red howler monkeys^80^; but see wolves^81^ and Australian magpies^82^). While the latter species use choruses to mediate aggressive inter-group encounters, male *S. bilineata* sing to mediate aggressive male-male interactions within colonies^38,39,50^ and the resulting dawn chorus is most probably a by-product of intense male-male competition in the day-roosts and not the result of coordinated communal signalling. Nevertheless, females might be able to extract valuable information about colony size and, thus, the number of potential mating partners^65,66^ from male dawn choruses.

We can only speculate whether singing in *S. bilineata* has predation costs, as has been shown for vocalizing insects, anurans and birds^83,84^. Since single territorial songs and, in particular, dawn choruses are conspicuous and widely audible displays, it is conceivable that they could attract acoustically orienting predators such as owls or coatis. Moreover, we have anecdotal evidence that female *S. bilineata* are extremely reluctant to settle in a known day-roost at dawn when no males are singing from that particular roost. Territorial songs may function as an ‘all-clear’ signal to females since singing males, being loud, stationary, and exposed, could indicate that no predators are currently around.

The dawn chorus of *S. bilineata* also encoded information on colony identity. This in is line with findings from group-living birds and mammals which deliver communal choruses in the context of territorial defence (laughing kookaburras^63^, green woodhoopoes^85^, and wolves^64^). However, we currently do not know whether conspecific bats make use of the colony signature encoded in territorial chorusing. Since the dawn chorus of a particular colony is always broadcast from the same location, spatial memory alone would suffice to distinguish different colonies. Nevertheless, it is interesting that not only single territorial songs^52^ but also whole choruses, composed of overlapping territorial songs from multiple males, encode information on colony identity.

Male territorial songs from different regions differ in their acoustic properties. While these regional differences have already been shown on a small spatial scale^53^ (up to 20 km distance between colonies), our study confirms this result on a much larger spatial scale, i.e. the population level (up to 1,120 km distance between different colonies). Female *S. bilineata* strongly prefer territorial songs from the local population over territorial songs from different foreign populations. This finding corresponds to female preferences for familiar male song in birds, e.g. in song sparrows^21^, brown-headed cowbirds^22^ and zebra finches^23^. Female preference for familiar song could simply be a by-product of species recognition mechanisms (reviewed in ref.^86^). However, it could also be a means of finding males with specific adaptations to the local habitat (reviewed in ref.^87^) or genetically high quality males because accurate imitation of local song indicates high learning abilities, which should be impaired in low quality, nutritional stressed birds^88–90^. At present, we do not know the functional significance of the preference for local songs observed in *S. bilineata* females but we argue that females’ production and perception learning are the mechanisms for this preference. Both male and female *S. bilineata* learn to produce territorial songs during ontogeny by imitating singing adult males^54^, even though only males produce territorial songs as adults^48^. Therefore, female production learning, in addition to female perception learning (e.g. shown for zebra finches^23,91^), most likely contributes to the formation of a mental template for local territorial songs heard during ontogeny.

In conclusion, our study provides strong experimental evidence that bat song is an important communication signal which elicits phonotaxis in female receivers. As a taxon, bats are extremely promising for comparative studies of mammalian song since there are over twenty known singing species from five different families^36^, and more are currently being described. Since bats have very diverse social systems (reviewed in ref.^92^) and natural histories (reviewed in ref.^93^), studies on the functions of male song under different selection pressures would be very promising. Moreover, bats are among the few mammals capable of vocal production learning (reviewed in ref.^94^). A phylogenetically broad comparison between learned birdsong and learned mammalian song could deepen our current knowledge of the physiological and neural mechanisms responsible for song production, perception and learning, and provide valuable insight into how song has evolved.

## Methods

### Sound recordings

Single territorial songs and whole song choruses were recorded with a high-quality recording set-up (500 kHz sampling rate and 16 bit depth resolution) consisting of an ultrasonic microphone (Avisoft USG 116Hme with condenser microphone CM16; frequency range 1–200 kHz) connected to a small computer (Lenovo S21e or Dell Venue 8) running the software Avisoft-Recorder (v4.2.05, R. Specht, Avisoft Bioacoustics, Glienicke, Germany). Recordings were conducted at dawn and dusk because the majority of territorial songs are produced during this time of day^48^. Single territorial songs were recorded at three different regions, namely Barro Colorado Island in Panama (region 1; 2010–2012), Curú in Costa Rica (region 2; 2010 and 2013) and Santa Rosa in Costa Rica (region 3; 2010 and 2014). We recorded nine males per region and selected ten territorial songs with excellent signal-to-noise ratio per male for subsequent acoustic analyses (assessing regional song differences) and playback experiments. Songs were filtered for background noise and normalized to 100%. Territorial song choruses were recorded in 2015 on Barro Colorado Island (region 1) using six day-roost colonies that differed in the number of singing males. Small colonies (N = 3) had 1–2 singing males, while large colonies (N = 3) had 3–4 singing males each. We analysed territorial song choruses to test whether they encoded a colony-specific vocal signature and information on colony size (large vs. small).

While single territorial songs were recorded with an optimal signal-to-noise ratio in mind (in the majority of cases, we recorded at unobstructed distances of four meters or less since the bats were well habituated to human observers), territorial song choruses were recorded from a greater distance to capture the overall acoustic impression of a colony with multiple singing males (Fig. 1). We recorded song choruses at a distance of seven meters and 14 meters, respectively, during a total of 35 recording sessions (5–6 sessions per colony, each lasting approx. 30 minutes). Subsequently, we extracted chorus excerpts of one second duration from our recordings for acoustical analyses (642 chorus excerpts from six colonies in total; equal amount per recording distance). Excerpts were selected whenever overlapping territorial songs (from at least two males) were detected. We selected 4–36 chorus excerpts per recordings session. All selected chorus excerpts were separated by at least 50 seconds, thus minimizing the temporal dependence of vocalizations produced in sequence. The distance between the microphone and the singing males was measured with a laser distance meter (Excelvan, California, USA) prior to each recording session. Our six focal colonies occupied day-roosts on the outside walls of buildings belonging to the Biological Station Barro Colorado Island (BCI) of the Smithsonian Tropical Research Institute (STRI) in Panama (9°9′17″ N, 79°51′53″ W).

### Amplitude measurements of territorial song choruses

While one person was conducting sound recordings, a second person was simultaneously measuring the amplitude of territorial song choruses using a sound level meter (CEL-246, Digital Logging Sound Level Meter Type 2, Casella CEL Inc., USA; measurement range: 30–130 dB SPL; frequency range: 20 Hz – 20 kHz; peak frequency of territorial songs: 14.5 kHz^49^). The sound level meter was used in a manual mode which allowed us to store measurements in an internal memory. Measurements for each of the six colonies were conducted at two distances (7 and 14 meters) during three different sessions each, with the exception of one colony, which was only measured during two sessions at 14 meter distance (35 sessions in total). For each colony, amplitude measurements were always conducted from the same position, so the sound attenuating obstacles, such as leaves and branches, between the singing bats and the sound level meter were comparable. Measurement sessions for the same colony were conducted on different days. Multiple measurements (N = 13) per session were averaged, resulting in a total of 35 amplitude measurements. Measurements of chorus amplitude ceased whenever other animals (crickets, katydids, cicadas, frogs, etc.) were vocalizing nearby to avoid confusion of signal amplitudes. We used the amplitude measurements of song choruses to test whether large colonies had larger chorus amplitudes than small colonies. We did not measure environmental background noise since all six colonies were in the same habitat in direct vicinity to one another.

### Estimation of signalling range for single territorial songs

Male *S. bilineata* produce territorial songs with an amplitude of 96 dB SPL at a distance of one meter^49^. To estimate the signalling range for single territorial songs, we used a formula originally developed for calculating the maximum detection distance of objects by bat echolocation calls^67^, which depends on atmospheric conditions, echolocation call frequency, dynamic range of the sonar system (i.e. the amplitude of echolocation calls and the receivers’ detection threshold) and target properties. Values for reflection loss (i.e. the fraction of energy reflected) and spreading (i.e. the loss due to energy spreading on the propagated way) were taken directly from^67^ and refer to an acoustic mirror target (reflection loss: −6, spreading: −20). Since the original formula calculates the maximum detection distance of objects by bat echolocation calls (which have to propagate forth to the target and back to the signaller), we multiplied the maximum detection distance by two to estimate the signalling range of territorial songs (since territorial songs do not have to propagate back to the signaller). We used a source level of 96 dB SPL and a detection threshold of 20 dB SPL (or 0 dB SPL when calculating the hypothetical maximum signalling range), resulting in a dynamic range of 76 dB SPL (or 96 dB SPL, respectively). For 20 kHz, we assumed an atmospheric attenuation of 0.27 dB SPL per meter (101325 Pa, 25 °C, 100% humidity), which is well suited for a Neotropical low-elevation site at dawn. We used a frequency of 20 kHz for our calculations since the formula was developed for frequencies equal to or above 20 kHz^67^, even though territorial songs have a lower frequency (median peak frequency of 14.5 kHz^49^). Therefore, our calculations most probably underestimated the actual signalling range of territorial songs. Moreover, we used two different detection thresholds for receivers: 20 dB SPL and 0 dB SPL (to calculate the hypothetical maximum signalling range). 20 dB SPL were estimated for echolocation pulses^67^ and may be too high for territorial songs (but there are currently no better estimates available for bat song) which is why we also reported the hypothetical maximum signalling range.

### Acoustic analyses of single territorial songs

We used the software Avisoft SASLab Pro (v.5.2.01; R. Specht, Avisoft Bioacoustics, Germany) for acoustic analyses. Spectrograms were created using a Hamming window with 1024-point fast Fourier transform and 87.5% overlap (frequency resolution: 488 Hz; time resolution: 0.256 ms). Territorial songs consist of different syllables that gradually merge from one syllable type into the next (Fig. 1a). Syllables were defined as a vocalization bout surrounded by silence^48^. Territorial songs end with composite syllables (with a pulsed and a tonal component) that are called “buzz syllables”. Since a previous study indicated that most information about the signaller is encoded in buzz syllables^52^, we measured three consecutive buzz syllables per territorial song. Acoustic parameters of buzz syllables were subsequently averaged (separately for pulsed and tonal parts) to minimize temporal dependence among syllables produced in direct succession. Overall, we analysed 810 buzz syllables belonging to 270 territorial songs produced by 27 different males from three regions. Since we wanted to assess regional differences in territorial songs, we averaged acoustic parameters of buzz syllables not only per song but also per male. This was necessary because territorial songs encode an individual signature^50,52,53^ which might have interfered with our analysis on regional song differences.

Even though territorial songs were multiharmonic, we used only the fundamental frequency (first harmonic) for automated parameter measurements in Avisoft SASLab Pro because it contained most of the sound energy. In total, we measured eight acoustic parameters for buzz syllables. For the pulsed part of buzz syllables, we measured the duration and the peak frequency at start and end of the syllable part. For the tonal part of buzz syllables, we measured the duration and the peak frequency at four different locations distributed equally over the tonal part.

### Acoustic analyses of territorial song choruses

We used a custom-made routine in the speech processing toolbox ‘voicebox’ in MATLAB (v. R2014a) to analyse territorial song choruses. In total, we analysed 642 chorus excerpts (of one second duration) from six colonies recorded at distances of seven and 14 meters (321 excerpts per recording distance) during 35 sessions. Since song choruses were composed of overlapping territorial songs from multiple males, it was not possible to extract classical acoustic parameters of single buzz syllables. Instead, we used an acoustic feature extraction technique based on linear-frequency cepstral coefficients (LFCC). LFCCs are spectral-based representations of entire signals, capturing most important features of signals in a compact form. This technique is widely used for human voice analysis and human speaker recognition (reviewed in refs^95,96^). We used the computed LFCCs as acoustic parameters in subsequent statistical analyses (see below). In total, we extracted five LFCCs to describe the acoustic properties of territorial song chorus excerpts.

### Playback experiments

We selected nine currently uninhabited but principally suitable day-roosts for our playback experiments. Five day-roosts were trees inside the forest, one was a tree at the forest edge, and three day-roosts were buildings surrounded by forest (see Fig. S1 in Supplementary Information for details). Dispersing females were born in 2014 and 2015, respectively, and could not have knowledge about these day-roosts since all of them were uninhabited since 2012. Adult females attracted to our playbacks could theoretically have had prior knowledge about the location of the currently uninhabited day-roosts. All playback sites were out of earshot from the next inhabited day-roost to prevent resident territorial males from counter-singing in response to our playbacks. We conducted one playback trial per site; the same sites were used for both experiments. All playbacks were conducted in August because during this time subadult females disperse form their natal colonies and immigrate into new colonies before first conception in December^46^.

Females are known to disperse within the area they were born in (in our study populations, we observed dispersal distances of up to three kilometres). The sex ratio of subadults in our study area in Panama was 1:1. This ratio was derived from census data of banded subadults in monitored roosts and from five years of capture data (2010–2015). The proportion of adult females to subadult females in our study area in Panama was 2.4:1. This proportion was derived from census data of banded individuals in monitored roosts (2014–2015). Therefore, an equal proportion of subadult males and females and a larger proportion of adult females than subadult females were exposed to our playbacks. We conducted each playback trial at dawn when the local population of territorial males started singing (at 6 a.m.). Territorial males sing while being perched in their day-roost while all other conspecifics (i.e. bachelor males without a territory, adult females, and subadults of both sexes) are still on the wing^48,49,52^.

In a first, preliminary playback experiment (August 2014), we tested whether territorial songs (recorded from the local population in 2010 and 2011) were suitable for eliciting phonotaxis in dispersing females. We used silence as a ‘control stimulus’ for two reasons: no other social vocalizations from *S. bilineata* are broadcasted at dawn and a previous study indicated that noise stimuli actively repel *S. bilineta* ^97^. We also refrained from using social vocalizations from sympatric bat species as a control since they are broadcasted at lower amplitudes than territorial songs and might have easily been masked by the ambient noise level at dawn. In each playback trial, we broadcasted two sound files (three minutes of territorial songs and three minutes of silence) in a balanced order per site. We broadcasted ten different territorial songs from one male per playback trial. Each territorial song was repeated once, resulting in 20 songs in three minutes. Silent intervals between songs ranged from 6–10 seconds, mimicking natural song intervals; the order of songs was randomized. We used a different stimulus donor male for each playback site. The nine donor males were unfamiliar to the dispersing females because they were not present in our population any more. A three-minute pause between the two sets of stimuli (songs and silence) allowed us to free any captured bat (see below) before the second set of stimuli were presented. In total, a playback trial had a duration of nine minutes (stimulus set 1 - pause - stimulus set 2). Playback stimuli (500 kHz sampling rate and 16-bit depth resolution) were broadcasted with an ultrasonic speaker (Avisoft UltraSoundGate Player BL Pro, single speaker version; 5–70 kHz ± 6 dB) connected to a laptop computer (Lenovo S21e) running the software Avisoft-Recorder (v. 4.2; R. Specht, Avisoft Bioacoustics, Glienicke, Germany). The amplitudes of playback stimuli were adjusted to 80 dB SPL at a distance of one meter. The speaker was positioned directly in the currently uninhabited day-roosts and mounted to the roosting surface with a rope or a pole. To capture bats inspecting the day-roost, we placed a small mist-net (Ultrathin Mist Nets M-14; Ecotone, Gdynia, Poland) directly in front of the speaker, with a distance of less than one meter. The mist-net was only 2.0 × 2.4 meters in size, thus assuring that we would only capture bats inspecting the day-roost, i.e. trying to land next to the speaker, and not commuting bats that were just passing by. Captured bats were extracted from the mist-net in the pause between the two sets of stimuli. They were kept in custom-made cages until the end of the playback trial. Cages were at least 30 meters away from the playback site to avoid any interference with the experiment. Subsequently, captured bats were sexed and their age (subadult or adult) was determined based on the ossification of the epiphyseal fusion. After marking them individually with coloured plastic bands on their forearms (AC Hughes® Ltd., UK, size XCL), all bats were released at the site of capture.

In the second playback experiment (August 2015), we tested whether dispersing females were more attracted to local territorial songs or to foreign territorial songs from two different regions (Curú and Santa Rosa in Costa Rica). The experimental design was similar to the first experiment, except that this time we broadcasted three sets of stimuli (with three-minute pauses between sets) in a balanced order per site. In total, playback trials had a duration of 12 minutes (stimulus set 1 - pause - stimulus set 2 - pause - stimulus set 3). The local territorial songs were the same as in the first playback experiment. The two sets of foreign territorial songs were recorded from nine different males per region (ten different songs per male). All 27 donor males were unfamiliar to the dispersing females in the area.

### Statistical Analyses

To test for acoustic differences between the territorial song choruses of large and small colonies, we performed separate Generalized Linear Mixed Models (GLMMs with colony size as fixed factor and colony ID as random factor; Gamma distribution with log link function) for chorus amplitude (N = 35 amplitude measurements) and the five extracted LFCCs (N = 642 analysed excerpts) as dependent variables. Separate GLMMs were conducted for data obtained at a recording distance of 7 meters and 14 meters, respectively. To test for a colony-specific signature in territorial song choruses, we performed two discriminant function analyses (DFAs) on the 7 m and 14 m data sets (with 321 chorus excerpts from 6 colonies each). The number of analysed chorus excerpts per colony remained the same in both DFAs to ensure comparability. Five uncorrelated LFCCs were simultaneously included in the DFA. We applied subset validation procedures in which ‘training’ sets (50% of chorus excerpts) were used to calculate the discriminant functions with which ‘test’ sets (remaining 50% of chorus excerpts) were classified. Chorus excerpts in the training sets were selected from different recording sessions than chorus excerpts in the test sets to prevent statistical overfitting. To test for a regional signature in single territorial songs, we performed a DFA with 27 males from three regions (9 males per region). Song parameter measurements were averaged per male (10 songs per male), so that each male constituted only one data point in the DFA. We simultaneously included eight acoustic parameters in the DFA, all of which were checked for multicollinearity. Since the sample size did not allow us to use a subset validation procedure, we used an ‘n-1’ cross-validation procedure instead which classified each male based on discriminant functions established with all males other than the one being classified. To analyse the data from both playback experiments (local song vs. silence; local song vs. two types of foreign song), we performed non-parametric tests for paired data, a Wilcoxon signed-rank test (playback 1) and a Friedman test (playback 2). All statistical tests were conducted in SPSS (v.20; IBM SPSS Statistics Chicago, IL, USA) and R (v.3.3.2; R Development Core Team 2008).

### Ethical standards

The process of acquiring data, protocols for capturing and handling bats, and experimental protocols for playbacks complied with the current laws of Panama and were conducted in accordance with the relevant guidelines and regulations. Our study was approved by the Smithsonian Tropical Research Institute and its Animal Care and Use Committee (ACUC). Permit number: ACUC 2013-1015-2016.

### Data Availability

The datasets generated and analysed during the current study are available from the corresponding author on reasonable request.

## Electronic supplementary material

Supplementary information
