# Supplementary material for: Bat songs as acoustic beacons - male territorial songs attract dispersing females
Source: Sci Rep. 2017 Oct 24;7:13918. doi: 10.1038/s41598-017-14434-5 (PMC5654967; doi:10.1038/s41598-017-14434-5)
Supplement: Supplementary file 1 — Supplementary information [file 41598_2017_14434_MOESM1_ESM.pdf]

SUPPLEMENTARY INFORMATION

Bat songs as acoustic beacons - male territorial songs attract dispersing females

Mirjam Knörnschild, Simone Blüml, Patrick Steidl, Maria Eckenweber, Martina Nagy

The supplementary information contains four tables and two figures.

Table S1: Number of captured female bats exhibiting phonotaxis to our playbacks.

Playback trial	1. playback (local song vs. silence)		2. playback (local song vs. foreign songs)		
	Local song	Silence	Local song	Foreign song 1	Foreign song 2
1	1 SA	0	1 SA	0	0
2	1 SA	0	1SA + 1A	0	0
3	1SA + 1A	0	1SA + 1A	0	0
4	1SA + 1A	0	1 SA	0	0
5	1 SA	0	1SA + 1A	0	1 SA
6	1 SA	0	1 SA	0	0
7	1SA + 1A	0	0	0	0
8	1 SA	0	2 SA	1 SA	0
9	2 SA	0	1 SA	0	0
total	10 SA + 3 A	0	9 SA + 3 A	1 SA	1 SA

SA stands for subadult female, A for adult female. No males were captured.

Table S2: Assessment of model fit of the discriminant function analysis (DFA) applied to single territorial songs recorded from 27 males at three different regions.

Function	Eigen-value	% of Variance	Test of Functions	Wilks λ	χ2	df	P
1	3.717	87.7	1 to 2	0.140	40.377	16	0.001
2	0.519	12.3	2	0.658	8.576	7	0.285

270 single territorial songs (ten songs per male, nine males per region, song parameters were averaged per male) were analysed and eight acoustic parameters were included simultaneously in the DFA. A cross-validation procedure (n-1) was used.

Table S3: Results of separate Generalized Linear Mixed Models with five derived acoustic parameters (LFCC 1 - 5) at two recording distances as dependent variables, colony size as fixed factor (large vs. small) and colony ID as random factor (colony 1 - 6).

Acoustic parameter	Recording distance	AIC	t	P
LFCC 1	7 m	776.4	-0.794	0.427
LFCC 2	7 m	60.5	-0.524	0.600
LFCC 3	7 m	-294.7	-2.156	0.031
LFCC 4	7 m	65.5	-1.586	0.113
LFCC 5	7 m	-151.6	-0.786	0.432
LFCC 1	14 m	197.5	0.026	0.979
LFCC 2	14 m	-273.2	0.143	0.886
LFCC 3	14 m	-951.0	-0.082	0.935
LFCC 4	14 m	-917.1	0.016	0.987
LFCC 5	14 m	-643.0	-0.240	0.810

A Gamma distribution with log link function was used for the GLMMs.

Table S4: Assessment of model fit of the discriminant function analyses (DFAs) applied to territorial song chorus excerpts recorded at distances of seven and 14 meters.

Analyses	Function	Eigen-value	% of Variance	Test of Functions	Wilks λ	χ2	df	P
DFA 1 (7 m)	1	1.896	49.1	1 to 5	0.089	371.583	25	< 0.0001
DFA 1 (7 m)	2	1.377	35.7	2 to 5	0.257	208.338	16	< 0.0001
DFA 1 (7 m)	3	0.488	12.7	3 to 5	0.612	75.434	9	< 0.0001
DFA 1 (7 m)	4	0.095	2.5	4 to 5	0.910	14.394	4	0.0006
DFA 1 (7 m)	5	0.003	0.1	5	0.997	0.521	1	0.4710
DFA 2 (14 m)	1	0.551	63.5	1 to 5	0.483	111.754	25	< 0.0001
DFA 2 (14 m)	2	0.244	28.1	2 to 5	0.749	44.421	16	< 0.0001
DFA 2 (14 m)	3	0.058	6.7	3 to 5	0.931	10.966	9	0.278
DFA 2 (14 m)	4	0.015	1.7	4 to 5	0.985	2.282	4	0.684
DFA 2 (14 m)	5	0	0	5	1.000	0.001	1	0.982

Two separate DFAs were calculated (for chorus excerpts recorded at 7m and 14m distance). Number of chorus excerpts (N=321) and derived acoustic parameters (LFCC 1-5) were similar for both DFAs. Subset validation procedures (50% of excerpts in the trainings and test sets, respectively) were used.

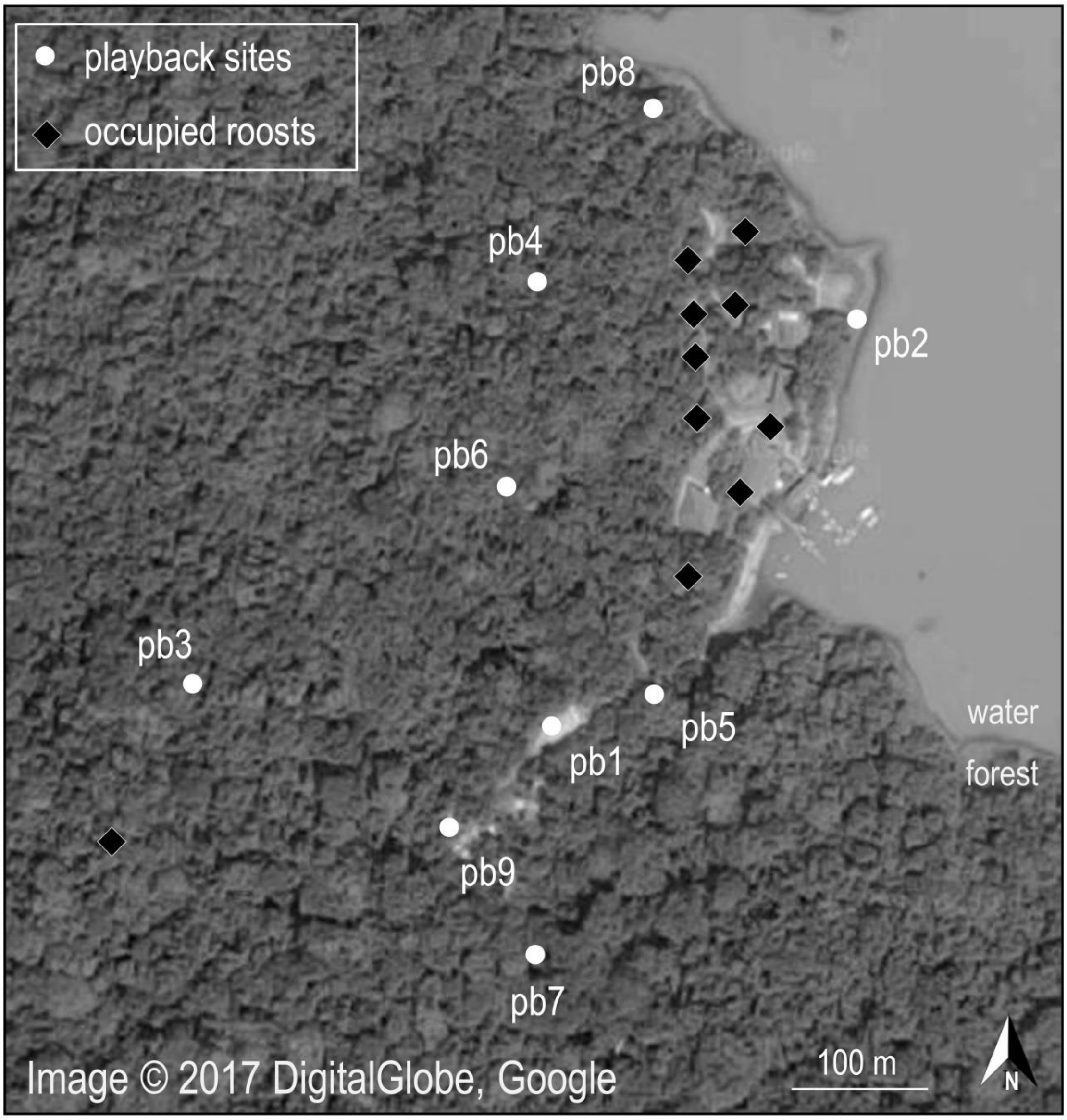

Figure S1: Map of study area depicting the location of playback sites (white circles) and known occupied day-roosts (black diamonds). Most day-roosts were situated on the outside walls of buildings belonging to the Biological Station Barro Colorado Island of the Smithsonian Tropical Research Institute (STRI) in Panama; one day-roost was inside a hollow tree in the forest. Numbers next to white circles (pb1-pb9) indicate the order in which sites were used for playback experiments. The air-line distance between playback sites and occupied day-roosts ranged between 100-300 meters. We carefully selected playback sites that were out of earshot from day-roosts. The signalling range of territorial songs can be up to 184 meters in open habitats but the dense forest surrounding our playback sites attenuates sound, thus reducing the signalling range of territorial song playbacks. The sex ratio of subadults in our study area was 1:1. The proportion of adult females to subadult females in our study area was 2.4:1. Thus, an equal proportion of subadult males and females and a larger proportion of adult females than subadult females were exposed to our playbacks.
Image Data: 2017 DigitalGlobe, Google.

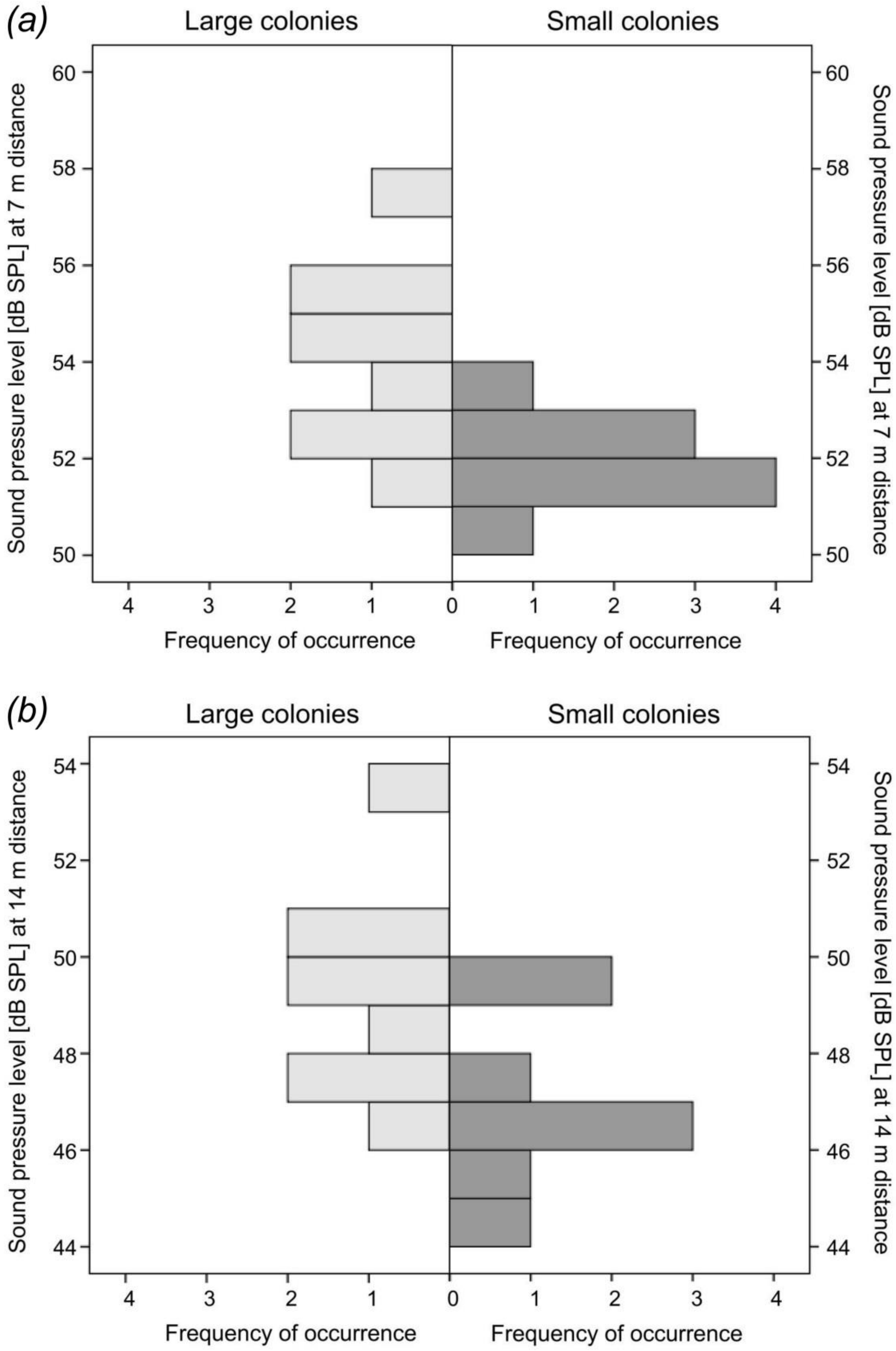

Figure S2: Amplitude of territorial song choruses recorded at a distance of 7 meters (a) and 14 meters (b). The same six colonies (three small colonies with 1-2 singing males and three large colonies with 3-4 singing males) were recorded at both distances. At 7 meters recording distance, each colony was sampled during three independent recording sessions. At 14 meters recording distance, one large colony was only sampled during two sessions, all other colonies during three sessions. Mean values for each recording session are shown. Please note the different ranges of dB SPL scales for (a) and (b).
